# Evaluation of Dietary Niacin and New-Onset Hypertension Among Chinese Adults

**DOI:** 10.1001/jamanetworkopen.2020.31669

**Published:** 2021-01-06

**Authors:** Zhuxian Zhang, Mengyi Liu, Chun Zhou, Panpan He, Yuanyuan Zhang, Huan Li, Qinqin Li, Chengzhang Liu, Xianhui Qin

**Affiliations:** 1 National Clinical Research Center for Kidney Disease, State Key Laboratory for Organ Failure Research, Division of Nephrology, Nanfang Hospital, Southern Medical University, Guangzhou, China; 2 Guangdong Provincial Key Laboratory of Renal Failure Research, Guangzhou Regenerative Medicine and Health Guangdong Laboratory, Guangzhou, China; 3 Institute of Biomedicine, Anhui Medical University, Hefei, China

## Abstract

**Importance:**

The relationship of dietary niacin intake with the risk of hypertension remains unknown.

**Objective:**

To determine the prospective association between dietary niacin intake and new-onset hypertension, and examine factors that may modify the association among Chinese adults.

**Design, Setting, and Participants:**

This nationwide cohort study of 12 243 Chinese adults used dietary intake data from 7 rounds of the China Health and Nutrition Survey. Dietary intake was measured by 3 consecutive 24-hour dietary recalls from participants in combination with a weighing inventory taken over the same 3 days at the household level. Statistical analysis was conducted from May 2020 to August 2020.

**Exposures:**

Dietary intake.

**Main Outcomes and Measures:**

The study outcome was new-onset hypertension, defined as systolic blood pressure 140 mm Hg or greater and/or diastolic blood pressure 90 mm Hg or greater, diagnosis by physician, or current antihypertensive treatment during the follow-up.

**Results:**

The mean (SD) age of the study population was 41.2 (14.2) years, and 5728 (46.8%) of participants were men. The mean (SD) dietary niacin intake level was 14.8 (4.1) mg/d. A total of 4306 participants developed new-onset hypertension during a median (interquartile range) follow-up duration of 6.1 (3.6-11.3) years. When dietary niacin was assessed in quartiles, the lowest risk of new-onset hypertension was found in participants in quartile 3 (14.3 to <16.7 mg/d; adjusted hazard ratio, 0.83; 95% CI, 0.75-0.90) compared with those in quartile 1 (<12.4 mg/d). Consistently in the threshold analysis, for every 1 mg/d increase in dietary niacin, there was a 2% decrease in new-onset hypertension (adjusted HR, 0.98; 95% CI, 0.96-1.00) in those with dietary niacin intake less than 15.6 mg/d, and a 3% increase in new-onset hypertension (adjusted HR, 1.03; 95% CI, 1.02-1.04) in participants with dietary niacin 15.6 mg/d or greater. Based on these results, there was a J-shaped association between dietary niacin intake and new-onset hypertension in the general population of Chinese adults, with an inflection point at 15.6 mg/d and a minimal risk at 14.3 to 16.7 mg/d (quartile 3) of dietary niacin intake.

**Conclusions and Relevance:**

The results of this study provide some evidence for maintaining the optimal dietary niacin intake levels for the primary prevention of hypertension.

## Key Points

**Question:**

Is there an association between dietary niacin intake and the risk of new-onset hypertension?

**Findings:**

In this nationwide cohort study, a J-shaped association was found between dietary niacin intake and new-onset hypertension in Chinese adults, with an inflection point at about 15.6 mg/d and minimal risk between 14.3 and 16.7 mg/d of dietary niacin intake.

**Meaning:**

The results of this study provide evidence that maintaining optimal dietary niacin intake levels may support the primary prevention of hypertension.

## Teaser

This cohort study of a large nationwide database of Chinese adults assesses the association between dietary niacin intake and the risk of new-onset hypertension.

## Introduction

Hypertension is a leading cause of noncommunicable diseases, mortality, and disability worldwide.^1,2,3^ Approximately one-third of the adult population, or more than 300 million people, had hypertension in China between 2014 and 2015.^4,5^ Therefore, there is an urgent need to identify high-risk individuals and develop effective primary prevention strategies to reverse the rapidly rising trend of hypertension.

Niacin, also known as nicotinic acid or vitamin B_3_, is a vitamin precursor of nicotinamide adenine dinucleotide and is therefore essential for energy metabolism and redox reactions.^6^ Studies have shown that niacin supplementation may regulate abnormal lipid metabolism, improve endothelial function, and have antioxidant and anti-inflammatory properties.^7^ Nevertheless, excessive niacin is also engaged in numerous pathologies, including insulin resistance and elevated homocysteine (HCY) levels.^8,9^ Several randomized clinical trials have assessed the effect of niacin supplementation on blood pressure (BP), but the results were inconsistent.^10,11,12,13,14,15^ Of note, these trials mainly examined the effects of relatively high niacin supplementation in high-risk populations rather than the effects of dietary niacin derived from foods in general populations. The dietary sources of niacin mainly include cereals and cereal products, meat and meat products, and vegetables.^16^ However, to date, research on the association between dietary niacin intake and hypertension is limited, and the prospective association between dietary niacin intake and incident hypertension risk remains unknown in the general population.

To address these knowledge gaps, our present study aimed to investigate the prospective association between dietary niacin intake and the risk of new-onset hypertension and to examine factors that may modify the association in the general population using data from the nationwide China Health and Nutrition Survey (CHNS).

## Methods

### Study Design and Population

Details on the study design and major results of the CHNS have been described previously.^17,18,19^ In brief, the CHNS is an ongoing multipurpose longitudinal open cohort study established in 1989, with follow up scheduled for every 2 to 4 years. A multistage, random cluster approach was used to sample the study population from 9 provinces (Heilongjiang [enrolled in 1997], Liaoning, Shandong, Henan, Jiangsu, Hubei, Hunan, Guizhou, and Guangxi) and 3 of China’s largest autonomous cities (Beijing, Shanghai, and Chongqing [all enrolled in 2011]). The CHNS rounds were conducted in 1989, 1991, 1993, 1997, 2000, 2004, 2006, 2009, 2011, and 2015. By 2011, the CHNS included 12 provinces and autonomous cities and 288 communities; the provinces included in the CHNS constituted 47% of China’s population.^20^

We conducted a prospective cohort study based on 7 rounds of the CHNS data from 1997 to 2015. We first excluded participants who were pregnant, younger than 18 years, or with missing BP data. Among the remaining participants, those who were surveyed in at least 2 study rounds (15 774 participants; 61 612 person-waves) were included, and the first survey round was considered as baseline. The included population did not differ in most of the baseline characteristics from those not included (14 888 person-waves) (eFigure 1, eTable 1 in the Supplement). Of the 15 774 participants, we further excluded participants with hypertension (defined as having systolic blood pressure [SBP] ≥140 mm Hg and/or diastolic blood pressure [DBP] ≥90 mm Hg, previous diagnosis by physician, or currently receiving antihypertensive treatment) at the time of the first survey. Furthermore, participants with missing dietary niacin data or with extreme dietary energy data (for men, >8000 or <800 kcal/d; women, >6000 or <600 kcal/d) were also excluded. Overall, a total of 12 243 participants were enrolled in the final analysis (eFigure 1 in the Supplement).

The institutional review boards of the University of North Carolina at Chapel Hill, the National Institute of Nutrition and Food Safety, and the Chinese Center for Disease Control and Prevention approved the study. Each CHNS participant provided their written informed consent. The data, as well as study materials that support the findings of this study, are available at the CHNS website.^21^ This study followed the Strengthening the Reporting of Observational Studies in Epidemiology (STROBE) reporting guideline.

### Dietary Nutrient Intakes

Both individual and household-level dietary data in the CHNS were collected by trained nutritionists through face-to-face interviews in each survey round. Individual diet was repeatedly assessed by 3 consecutive 24-hour dietary recalls at the individual level in combination with a weighing inventory over the same 3 days at the household level.^22^ The 3 consecutive days were randomly allocated from Monday to Sunday and are almost equally balanced across the 7 days of the week for each sampling unit. Nutrient intakes were calculated using the China food composition tables. Specifically, our analysis did not include the dietary data from the 1989, 1991, or 1993 waves because the food codes in those data sets did not match the food codes in the composition tables (the matching codes were not publicly released). The accuracy of 24-hour dietary recall designed to assess energy and nutrient intake has been validated.^22^ In the analyses, 3-day average intakes of dietary macronutrients and micronutrients in each round were calculated.

In this study, we evaluated the energy-adjusted nutrient intake for dietary niacin using residual method.^23^ Cumulative intake values of each nutrient were calculated for each participant using all results up to the last visit prior to the date of new-onset hypertension (or all results for participants without new-onset hypertension) to represent long-term dietary intake and minimize within-person variation.

### Blood Pressure Measurements

Seated blood pressure measurements were obtained by trained research staff after the patients had rested for 5 minutes using a mercury manometer, following the standard method and with appropriately sized cuffs at each follow-up survey. Triplicate measurements on the same arm were taken in a quiet and bright room. The mean SBP and DBP of the 3 independent measures were used in analysis.

### Assessment of Covariates

Information on age, sex, urban or rural residence, region, education level (eg, illiteracy, primary school, middle school, and ≥high school), occupation (eg, farmer, worker, unemployed, and others), and smoking and drinking status were obtained from the questionnaires at each follow-up survey. Smoking was defined by whether participants had ever smoked cigarettes (including hand-rolled or device-rolled), and drinking was defined by whether participants had ever drunk beer or any other alcoholic beverage. Height, weight, and waist and hip circumference were measured following a standard procedure with calibrated equipment. Body mass index (BMI) was calculated as weight in kilograms divided by height in meters squared.

The questionnaire on physician-diagnosed hypertension and antihypertensive treatment included the following questions: “(1) Has a doctor ever told you that you suffer from high blood pressure? If yes, (2) for how many years have you had it? and (3) are you currently taking anti-hypertension drugs?” In China, the clinical diagnosis and treatment of hypertension were mainly according to the Chinese Guidelines for Prevention and Treatment of Hypertension (1999, 2005, 2010, and 2018 versions). In all versions, hypertension was defined as a clinical SBP of 140 mm Hg or greater and/or DBP 90 mm Hg or greater without the use of antihypertensive medications. Overall, all the physicians used the same criteria for the clinical diagnosis and treatment of hypertension during the follow-up period.

### Study Outcome

The study outcome was new-onset hypertension, defined as mean SBP≥140 mm Hg and/or mean DBP≥90 mm Hg, diagnosis by physician, or current antihypertensive treatment during the follow-up. When a participant was first identified with new-onset hypertension in a follow-up survey, the middle date between this and the nearest survey before was used to calculate the follow-up time. For those free of hypertension in all follow-up surveys, the last survey date was used to calculate the follow-up time.

### Statistical Analysis

We assumed that the annual incident hypertension rate of Chinese adults with low dietary niacin was about 6% (with a type I error rate of .05), and so an enrollment of approximately 3000 participants in each group stratified by dietary niacin intake (eg, low, medium, high) would be necessary to provide more than 80% power to observe hazard ratios (HRs) of 1.2 or more for the comparison between low and high vs medium dietary niacin group during a follow-up period of about 6 years. Thus, a sample size of about 10 000 would be required.

Statistical analysis was conducted from May 25, 2020, to August 6, 2020. The population characteristics are presented as mean (SD) for continuous variables and proportions for categorical variables by quartiles of dietary niacin. The differences in population characteristics were compared using ANOVA tests or χ^2^ tests.

The association of dietary niacin intake with new-onset hypertension were estimated using Cox proportional hazards models before and after adjustments for age, sex, BMI, smoking status, SBP, DBP, region, education, and occupation, as well as energy intake and sodium to potassium (Na/K) intake ratio. Threshold analysis in the association of dietary niacin intake with the study outcome was conducted with a 2-piecewise Cox regression model using a smoothing function. The threshold level (ie, inflection point) was determined using a likelihood-ratio test and bootstrap resampling methods.

Furthermore, possible modifications of the association between dietary niacin and new-onset hypertension were evaluated for the following variables: age (<40 [median] vs ≥40 years), sex, BMI (<25 vs ≥25), waist to hip ratio (<0.85 [median] vs ≥0.85), smoking status, drinking status, SBP (<120 vs ≥120 mm Hg), sodium to potassium intake ratio (<2.8 [median] vs ≥2.8), potassium (<1.4 [tertile 1] vs ≥1.4 g/d), sodium (<3.7 [tertile 1] vs ≥3.7 g/d), fat (<70.9 [median] vs ≥70.9 g/d), protein (<65.4 [median] vs ≥65.4 g/d), carbohydrate (<305.9 [median] vs ≥305.9 g/d), energy (<2162.0 [median] vs ≥2162.0 Kcal/d), fruit intake (0 vs >0 g/d) and vegetable intake (<356.4 [median] vs ≥356.4 g/d), residence (urban vs rural), and education level (≤primary school vs ≥middle school). Heterogeneity across subgroups was assessed by Cox proportional hazards models, and interactions between subgroups and dietary niacin intake were examined by likelihood ratio testing.

A 2-tailed *P* < .05 was considered to be statistically significant in all analyses. R software, version 3.6.1 (R Project for Statistical Computing) was used for all data analyses.

## Results

### Study Participants and Baseline Characteristics

Our study included 12 243 participants with complete dietary niacin intake measurements from the CHNS (eFigure 1 in the Supplement). The mean (SD) age of the study population was 41.2 (14.2) years, and 5728 (46.8%) of the participants were men. The mean (SD) and median (interquartile range [IQR]) of dietary niacin intake were 14.8 (4.1) and 14.3 (12.4-16.7) mg/d (to convert niacin to μmol/d, multiply by 8.123), respectively. Baseline characteristics of study participants are presented by quartiles of dietary niacin in Table 1. Participants with higher dietary niacin intake had lower BMI, SBP, and DBP; lower percentages of residence in east and central regions; higher percentages of urban residence, higher education levels, and higher intake of fat, protein, potassium, and fruit and vegetables; lower intake of energy, carbohydrates, and sodium; and a lower sodium to potassium intake ratio. They were also younger and more likely to be men, smokers, and drinkers and less likely to be farmers (Table 1).

**Table 1.** Population Characteristics by Quartiles of Dietary Niacin Intake

Variable	Participants, No. (%)				*P* value
	Q1 (<12.4 mg/d) (n = 3061)	Q2 (12.4 to <14.3 mg/d) (n = 3060)	Q3 (14.3 to <16.7 mg/d) (n = 3061)	Q4 (≥16.7 mg/d) (n = 3061)	
Men	1340 (43.8)	1317 (43.0)	1399 (45.7)	1672 (54.6)	<.001
Age, mean (SD), y	41.5 (14.6)	42.0 (14.3)	40.7 (14.0)	40.5 (13.7)	<.001
Blood pressure, mean (SD), mm Hg					
Systolic	114.5 (11.3)	113.8 (11.5)	113.1 (11.5)	114.2 (11.4)	<.001
Diastolic	74.3 (7.9)	74.3 (7.9)	73.6 (7.8)	74.4 (7.8)	<.001
BMI, mean (SD)	22.7 (3.1)	22.2 (3.0)	22.2 (3.0)	22.5 (3.1)	<.001
Waist to hip ratio, mean (SD)	0.9 (0.1)	0.8 (0.1)	0.8 (0.1)	0.9 (0.1)	<.001
Smoking status	882 (28.9)	853 (28.1)	920 (30.2)	1053 (34.5)	<.001
Drinking status	972 (32.1)	931 (30.9)	1019 (33.7)	1242 (40.9)	<.001
Urban residence	926 (30.3)	940 (30.7)	1170 (38.2)	1379 (45.1)	<.001
Region					
East and central	2187 (71.4)	1505 (49.2)	1398 (45.7)	1421 (46.4)	<.001
Northeast and north	525 (17.2)	779 (25.5)	614 (20.1)	571 (18.7)	
Southwest and south	349 (11.4)	776 (25.4)	1049 (34.3)	1069 (34.9)	
Occupation					
Farmer	1231 (40.8)	1328 (44.0)	1097 (36.0)	728 (24.0)	<.001
Worker	333 (11.0)	304 (10.1)	382 (12.6)	441 (14.5)	
Unemployed	819 (27.2)	721 (23.9)	719 (23.6)	790 (26.0)	
Other	631 (20.9)	668 (22.1)	845 (27.8)	1076 (35.5)	
Education					
Illiteracy	684 (22.8)	644 (21.6)	517 (17.2)	369 (12.2)	<.001
Primary school	604 (20.2)	637 (21.4)	599 (19.9)	503 (16.6)	
Middle school	1021 (34.1)	995 (33.4)	992 (33.0)	1008 (33.3)	
≥High school	685 (22.9)	705 (23.6)	902 (30.0)	1143 (37.8)	
Self-report diabetes	35 (1.2)	35 (1.2)	37 (1.2)	47 (1.6)	.47
Dietary intake					
Energy, mean (SD), Kcal/d	2276.2 (578.7)	2162.7 (519.8)	2120.6 (485.7)	2207.0 (566.2)	<.001
Fat, mean (SD), g/d	75.6 (32.6)	69.5 (28.7)	72.7 (27.1)	80.9 (30.4)	<.001
Carbohydrate, mean (SD), g/d	334.9 (110.4)	321.8 (101.3)	301.1 (94.4)	292.7 (100.7)	<.001
Protein, mean (SD), g/d	64.1 (18.7)	62.6 (17.1)	65.4 (15.8)	77.0 (24.5)	<.001
Sodium, mean (SD), g/d	5.5 (3.3)	5.0 (3.1)	4.7 (2.6)	4.9 (3.0)	<.001
Potassium, mean (SD), g/d	1.6 (0.5)	1.6 (0.5)	1.7 (0.5)	1.9 (1.0)	<.001
Na:K ratio	3.8 (2.5)	3.3 (2.1)	3.0 (1.7)	2.7 (1.8)	<.001
Vegetables, mean (SD), g/d	329.0 (138.1)	361.3 (138.4)	385.9 (144.4)	418.9 (185.3)	<.001
Fruit intake	1201 (39.2)	1354 (44.2)	1510 (49.3)	1543 (50.4)	<.001

Abbreviations: BMI, body mass index (calculated as weight in kilograms divided by height in meters squared); Na:K, sodium to potassium.

SI conversion factor: To convert niacin to μmol/d, multiply by 8.123.

### Association Between Dietary Niacin Intake and New-Onset Hypertension

During a median (IQR) follow-up of 6.1 years (3.6-11.3 years), 4306 (45.0 per 1000 person-years) participants developed new-onset hypertension. Of these, 834 (19.4%) were diagnosed with hypertension by a physician, 533 (12.4%) reported use of antihypertensive treatment during follow-up, and 3955 (91.8%) had a new-onset mean SBP of 140 mm Hg or greater and/or a mean DBP of 90 mm Hg or greater during follow-up. Some of the patients met at least 2 of the 3 criteria.

**Figure 1.**
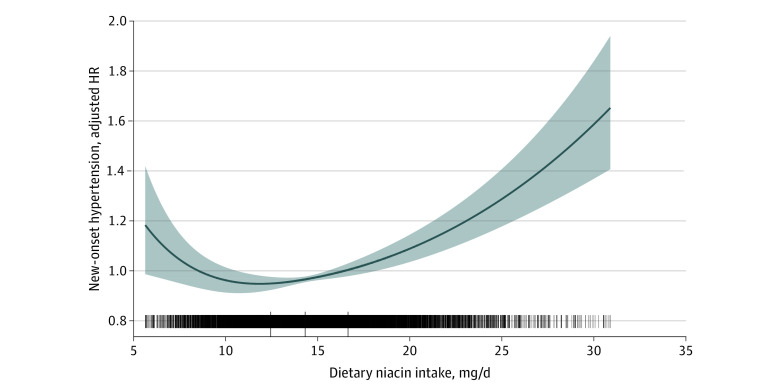
Dietary Niacin Intake and the Risk of New-Onset Hypertension The shaded area indicates 95% confidence intervals for adjusted hazard ratios (HR). The model was adjusted for age, sex, body mass index, smoking status, systolic blood pressure, diastolic blood pressure, region, education, and occupation, as well as energy intake and sodium to potassium intake ratio.

Overall, the association between dietary niacin and new-onset hypertension followed a J-shape (Figure 1). Accordingly, when dietary niacin intake was assessed in quartiles and compared with quartile 1 (<12.4 mg/d), the risk of new-onset hypertension was lower for quartile 2 (12.4 to <14.3 mg/d: HR, 0.90; 95% CI, 0.83-0.97; *P* = .01), quartile 3 (14.3 to <16.7 mg/d: HR, 0.70; 95% CI, 0.64-0.76; *P* < .001), and quartile 4 (≥16.7 mg/d: HR, 0.92; 95% CI, 0.85-1.00; *P* = .05) (Table 2). The lowest risk of new-onset hypertension was found in those in quartile 3. When combining quartiles in further exploratory analysis, a significantly higher risk of new-onset hypertension was found among participants in quartiles 1 and 2 (<14.3 mg/d: adjusted HR, 1.18; 95% CI, 1.09-1.28; *P* < .001) and in quartile 4 (≥16.7 mg/d: adjusted HR, 1.31; 95% CI, 1.20-1.44) compared with those in quartile 3 (14.3 to <16.7 mg/d) (Table 2).

**Table 2.** Dietary Niacin Intake and the Risk of New-Onset Hypertension Stratified by Quartiles and Combined Quartiles

Niacin intake, mg/d	Participants, No.	Events, No. (rate)^a^	Crude model		Adjusted model^b^	
			HR (95% CI)	*P* value	HR (95% CI)	*P* value
Quartiles						
Q1 (<12.4)	3061	1188 (51.7)	1 [Reference]		1 [Reference]	
Q2 (12.4 to <14.3)	3060	1166 (46.6)	0.90 (0.83-0.97)	.009	0.95 (0.87-1.04)	.27
Q3 (14.3 to <16.7)	3061	952 (36.2)	0.70 (0.64-0.76)	<.001	0.83 (0.75-0.90)	<.001
Q4 (≥16.7)	3061	998 (47.0)	0.92 (0.85-1.00)	.05	1.08 (0.99-1.19)	.09
Categories						
Q1-2 (<14.3)	6121	2354 (49.0)	1.36 (1.26-1.47)	<.001	1.18 (1.09-1.28)	<.001
Q3 (14.3 to <16.7)	3061	952 (36.2)	1 [Reference]		1 [Reference]	
Q4 (≥16.7)	3061	998 (47.0)	1.32 (1.21-1.44)	<.001	1.31 (1.20-1.44)	<.001

Abbreviations: HR, hazard ratio; Q, quartile.

SI conversion factor: To convert niacin to μmol/d, multiply by 8.123.

^a^ Incident rate is presented per 1000 person-years of follow-up.

^b^ Adjusted for age, sex, body mass index, smoking status, systolic blood pressure, diastolic blood pressure, region, education, and occupation, as well as energy intake and sodium to potassium intake ratio.

Consistently in the threshold analysis, for every 1 mg/d increase in dietary niacin there was a 2% decrease in new-onset hypertension (adjusted HR, 0.98; 95% CI, 0.96-1.00) in participants with dietary niacin less than 15.6 mg/d, and a 3% increase in new-onset hypertension (adjusted HR, 1.03; 95% CI, 1.02-1.04) in participants with dietary niacin 15.6 mg/d or greater (Table 3).

**Table 3.** Threshold Analyses of Dietary Niacin Intake on New-Onset Hypertension Using 2-Piecewise Regression Models

Niacin intake, mg/d	Crude model		Niacin intake, mg/d	Adjusted model^a^	
	HR (95% CI)	*P* value		HR (95% CI)	*P* value
<16.0	0.95 (0.93-0.96)	<.001	<15.6	0.98 (0.96-1.00)	.04
≥16.0	1.04 (1.03-1.05)	<.001	≥15.6	1.03 (1.02-1.04)	<.001

Abbreviation: HR, hazard ratio.

SI conversion factor: To convert niacin to μmol/d, multiply by 8.123.

^a^ Adjusted for age, sex, body mass index, smoking status, systolic blood pressure, diastolic blood pressure, region, education, and occupation, as well as energy intake and sodium to potassium intake ratio.

Moreover, further adjustments for waist to hip ratio, drinking status, sodium, and fruit and vegetable intake (eTable 2 in the Supplement), or excluding participants from the 3 autonomous cities (eTable 3 in the Supplement) did not substantially alter the association between dietary niacin and new-onset hypertension. Similar trends were also found for different components of new-onset hypertension, including hypertension diagnosed by a physician and participants who were using antihypertensive treatment during follow-up, and participants with mean SBP 140 mm Hg or greater and/or mean DBP of 90 mm Hg or greater during follow-up (eTable 4 in the Supplement).

### Stratified Analyses by Additional Factors

**Figure 2.**
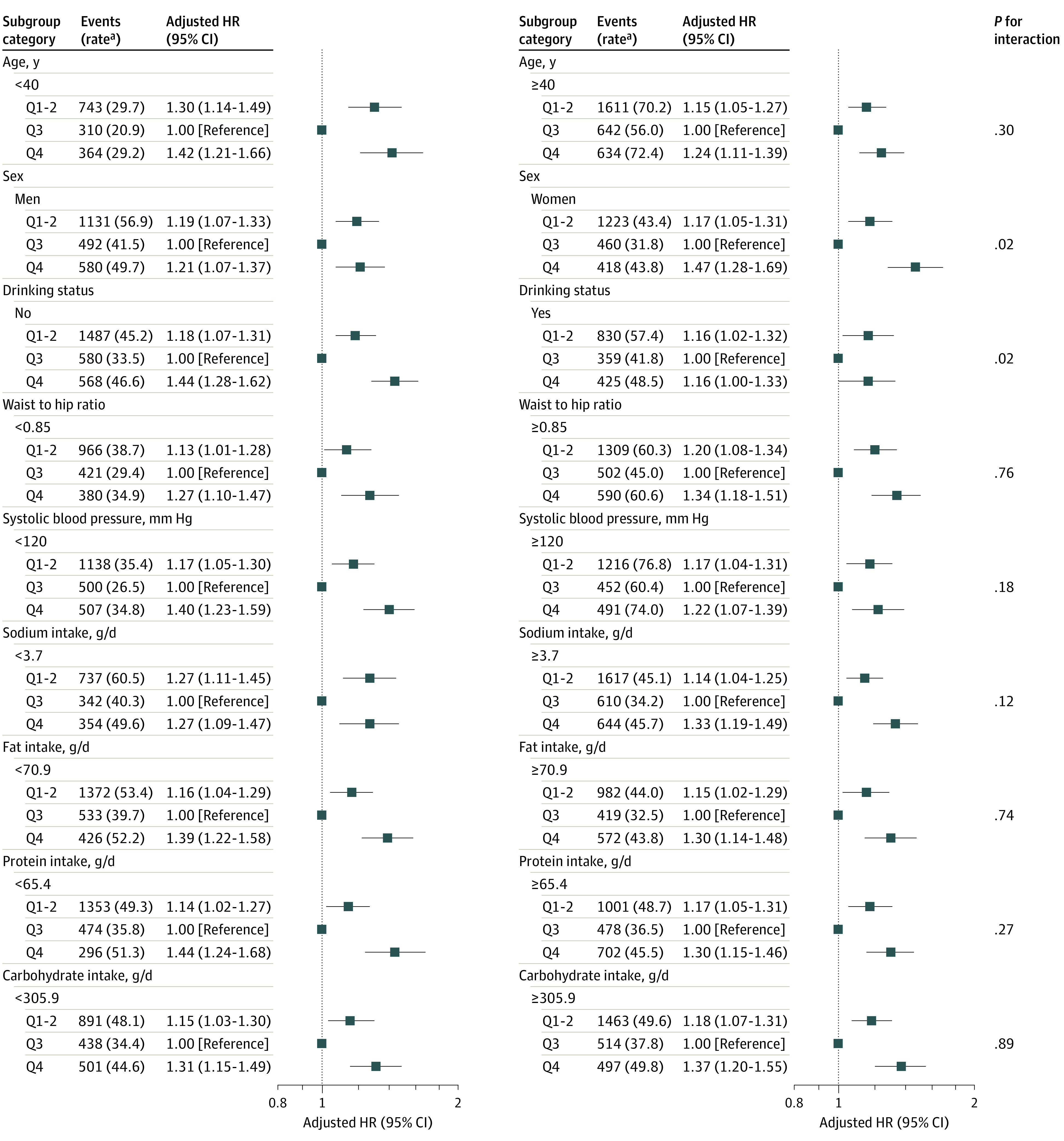
Stratified Analyses by Potential Modifiers of the Association Between Dietary Niacin Intake and New-Onset Hypertension ^a^Incident rate is presented per 1000 person-years of follow-up. The model was adjusted, if not stratified, for age, sex, body mass index, smoking status, systolic blood pressure, diastolic blood pressure, region, education, and occupation, as well as energy intake and sodium to potassium intake ratio.

We performed further stratified analyses to assess the association between dietary niacin (quartile 1-2 vs quartile 3 vs quartile 4) and the risk of new-onset hypertension in various subgroups (Figure 2; eFigure 2 in the Supplement). Overall, the J-shaped association between dietary niacin intake and new-onset hypertension was observed in all subgroups.

None of the variables, including age, sex, BMI, waist/hip ratio, smoking status, drinking status, SBP, sodium to potassium intake ratio, potassium, sodium, fat, protein, carbohydrate, energy, fruit and vegetable intake, residence, and education level significantly modified the association between dietary niacin and new-onset hypertension (Figure 2; eFigure 2 in the Supplement). Although the *P* values for interactions for sex, drinking status, and vegetable intake were lower than .05, these results may not have significant clinical implications given multiple testing and similar directionality of the associations (Figure 2; eFigure 2 in the Supplement).

## Discussion

In this large, national, longitudinal cohort study among general Chinese adults, we found a J-shaped association between dietary niacin intake and new-onset hypertension, with an inflection point at approximately 15.6 mg/d and minimal risk at 14.3 to 16.7 mg/d of dietary niacin.

The acute and chronic effects of niacin on blood pressure have been evaluated in several previous trials, which have reported inconsistent results.^10,11,12,13,14,15^ The original Coronary Drug Project revealed that no significant changes in BP were found in men with previous myocardial infarction over 5 to 8.5 years of niacin treatment (3.0 g/d).^10^ However, a post hoc analysis of the data in patients with metabolic syndrome found that treatment with niacin was associated with a reduction in BP of approximately 2 to 3 mm Hg compared with placebo at treatment year 1.^11^ At the same time, Kelly et al^14^ found that short-term niacin treatment (ie, 500 mg daily for 7 days, then 1 g daily for a further 7 days) did not significantly affect SBP or DBP. An 8-week niacin titration (1 g/d for 4 weeks, 2g/d for the remaining 4 weeks) study of 412 dyslipidemic patients also showed no significant change in BP from baseline.^12^ Nevertheless, Gadegbeku et al^15^ reported that acute niacin administration (1.4 g infusion for 60 min) may lower BP in patients with hypertension, but not in normotensive patients. Data from a longer and larger (ie, 24 wk with 1613 participants) study suggested that, compared with placebo, niacin therapy (1 g/d for 4 weeks, then 2 g/d for 20 weeks) in patients with dyslipidemia significantly decreased BP at both 4 and 24 weeks.^13^ Overall, these studies indicated that the association between niacin supplementation and BP remains uncertain. Of note, all of these studies focused on relatively high levels of niacin supplementation and did not have detailed information about dietary niacin intake. Although it is reported that nutrients obtained from foods and supplements may confer different health effects,^24^ to date the association between dietary niacin intake and hypertension has not been thoroughly investigated. The CHNS study provides an opportunity to evaluate the dose-response association between dietary niacin intake and the risk of new-onset hypertension in the general population, with comprehensive adjustments for a number of known covariables and a series of subgroup analyses.

Our study provides some new insights. First, among participants with dietary niacin of less than 15.6 mg/d, the risk of new-onset hypertension significantly decreased with the increment of dietary niacin intake. Niacin has been widely used clinically to regulate abnormalities in lipid/lipoprotein metabolism. Some studies have found that niacin alone or in combination can slow or reverse the progression of atherosclerosis in patients with hypercholesterolemia.^25,26^ Endothelial dysfunction is considered to be the initial phase in the development of arterial hypertension and atherosclerosis.^27^ Niacin has been shown to promote the production of endothelial nitric oxide, increase vasodilation, and improve endothelial dysfunction.^28,29^ Moreover, niacin also reduces endothelial oxidative stress via increasing the cellular content of nicotinamide adenine dinucleotide phosphate, reducing glutathione, and inhibiting reactive oxygen species generation in endothelial cells.^30,31^ Additionally, niacin can reduce the release of inflammatory markers such as lipoprotein-associated phospholipase A2 and high sensitivity C-reactive protein.^32,33^ Taken together, a plausible biological explanation for the niacin-hypertension association we observed may be that niacin can regulate abnormal lipid metabolism, improve endothelial function, and has potentially antioxidant and anti-inflammatory properties.^7^ However, further research on this mechanism is needed.

Second, the risk of new-onset hypertension significantly increased with the increment of dietary niacin intake in participants with dietary niacin of 15.6 mg/d or greater. Elevated HCY and insulin resistance may impair endothelial function and are identified as important risk factors for hypertension.^34,35^ It was reported that an increased nicotinamide load resulted in a significant increase in pulse pressure, which might be related to the fact that high niacin intake depletes the methyl pool, increases HCY generation and betaine consumption, and inhibits catecholamine degradation.^9,36^ In addition, a previous study found that treatment with niacin was related to increased insulin resistance as well.^14^

Of note, Table 1 shows that participants with lower dietary niacin (quartile 1 and 2) were older and had higher SBP and DBP levels, lower percentages of residence in urban and southern regions, and a higher sodium to potassium intake ratio. All these variables may partly explain the increased hypertension risk in participants with lower dietary niacin in the crude model in Table 2. As expected, with the increase of these variables in the adjusted models, the HR (ie, quartile 1-2 vs quartile 3) decreased gradually. We speculated that the change between the crude and adjusted models may be in part accounted for by the joint effect of these baseline characteristics. However, our results should be further confirmed in more studies.

### Limitations

Our study had several limitations. First, because this is an observational analysis, residual confounding cannot be completely eliminated, although data were adjusted for a series of confounders. Second, the biosynthesis of niacin from tryptophan was not included in our analysis. In general, 60 mg of tryptophan is equivalent to 1 mg of niacin through de novo synthesis.^37^ Nevertheless, this biosynthesis process does not occur in all tissues.^38^ In our stratified analysis, protein intake did not significantly modify the association between dietary niacin intake and new-onset hypertension. Third, we have no detailed information on dietary supplement use. However, data from the 2010–2012 China Nutrition and Health Surveillance study, a nationally representative cross-sectional study covering all 31 provinces, autonomous regions, and municipalities, showed that only 0.71%, 0.06%, and 0.2% of the Chinese population reported using nutrient supplements, multivitamins, and vitamin B supplements, respectively.^39^ Because of the low proportion of nutrient supplementation, especially vitamin B, we speculate that our findings may not be substantially changed by dietary supplement use. Fourth, because only 53 participants reported the use of special dietary patterns for the treatment of diabetes, and we lack information on circulating cholesterol levels in the present study, we could not examine the modifying effect of different dietary patterns and hypercholesterolemia. Other information was limited in our data source; the CHNS did not include clinic-based blood pressure measurements, and although the CHNS took place in different provinces and municipal cities that vary substantially in geography, economic development, public resources, and health indicators, the study participants could not represent the population of provinces or cities that were not included in the survey. Fifth, compared with those not included in the analysis, participants included seemed to be older and have a lower education level. However, all these variables were included in the regression models, and the stratified analysis further showed that age and education level did not materially modify the results. Sixth, our study was conducted among Chinese living in China—whether the observed findings can be extrapolated to other populations needs further investigation. Therefore, our results should be regarded as hypothesis generating. Further confirmation of our findings in more studies is essential.

## Conclusions

In summary, our study found a J-shaped association between dietary niacin intake and new-onset hypertension in the general population of Chinese adults, with an inflection point at about 15.6 mg/d and minimal risk observed at 14.3 to 16.7 mg/d of dietary niacin. If further confirmed, our data provide evidence for maintaining the optimal dietary niacin intake levels for the primary prevention of hypertension.

## Notes

Supplement. **eFigure 1.** Flow Chart of the Participants **eFigure 2.** Stratified Analyses by Potential Effect Modifiers for the Association Between Dietary Niacin Intake (Quartile 1-2 vs. Quartile 3 vs. Quartile 4) and New-Onset Hypertension **eTable 1.** Characteristics of the Included and Excluded Participants **eTable 2.** The Association Between Dietary Niacin Intake and the Risk of New-Onset Hypertension With Further Adjustment for Waist/Hip Ratio, Drinking Status, Sodium, Fruits and Vegetables Intake **eTable 3.** The Association Between Dietary Niacin Intake and the Risk of New-Onset Hypertension With Exclusion of Participants From the Three Autonomous Cities **eTable 4.** The Association Between Dietary Niacin Intake and Different Components of New-Onset Hypertension
