# Supplementary material for: Evaluation of Dietary Niacin and New-Onset Hypertension Among Chinese Adults
Source: JAMA Netw Open. 2021 Jan 6;4(1):e2031669. doi: 10.1001/jamanetworkopen.2020.31669 (PMC7788463; doi:10.1001/jamanetworkopen.2020.31669)
Supplement: Supplement. — eFigure 1. Flow Chart of the Participants eFigure 2. Stratified Analyses by Potential Effect Modifiers for the Association Between Dietary Niacin Intake (Quartile 1-2 vs. Quartile 3 vs. Quartile 4) and New-Onset Hypertension eTable 1. Characteristics of the Included and Excluded Participants eTable 2. The Association Between Dietary Niacin Intake and the Risk of New-Onset Hypertension With Further Adjustment for Waist/Hip Ratio, Drinking Status, Sodium, Fruits and Vegetables Intake eTable 3. The Association Between Dietary Niacin Intake and the Risk of New-Onset Hypertension With Exclusion of Participants From the Three Autonomous Cities eTable 4. The Association Between Dietary Niacin Intake and Different Components of New-Onset Hypertension [file jamanetwopen-e2031669-s001.pdf]

Supplemental Online Content

Zhang Z, Liu M, Zhou C, et al. Evaluation of dietary niacin and new-onset hypertension among Chinese adults. *JAMA Netw Open*. 2021;4(1):e2031669.
doi:10.1001/jamanetworkopen.2020.31669

eFigure 1. Flow Chart of the Participants

eFigure 2. Stratified Analyses by Potential Effect Modifiers for the Association Between Dietary Niacin Intake (Quartile 1-2 vs. Quartile 3 vs. Quartile 4) and New-Onset Hypertension

eTable 1. Characteristics of the Included and Excluded Participants

eTable 2. The Association Between Dietary Niacin Intake and the Risk of New-Onset Hypertension With Further Adjustment for Waist/Hip Ratio, Drinking Status, Sodium, Fruits and Vegetables Intake

eTable 3. The Association Between Dietary Niacin Intake and the Risk of New-Onset Hypertension With Exclusion of Participants From the Three Autonomous Cities

eTable 4. The Association Between Dietary Niacin Intake and Different Components of New-Onset Hypertension

This supplemental material has been provided by the authors to give readers additional information about their work.

© 2021 Zhang Z et al. *JAMA Network Open.*

eFigure 1. Flow chart of the participants

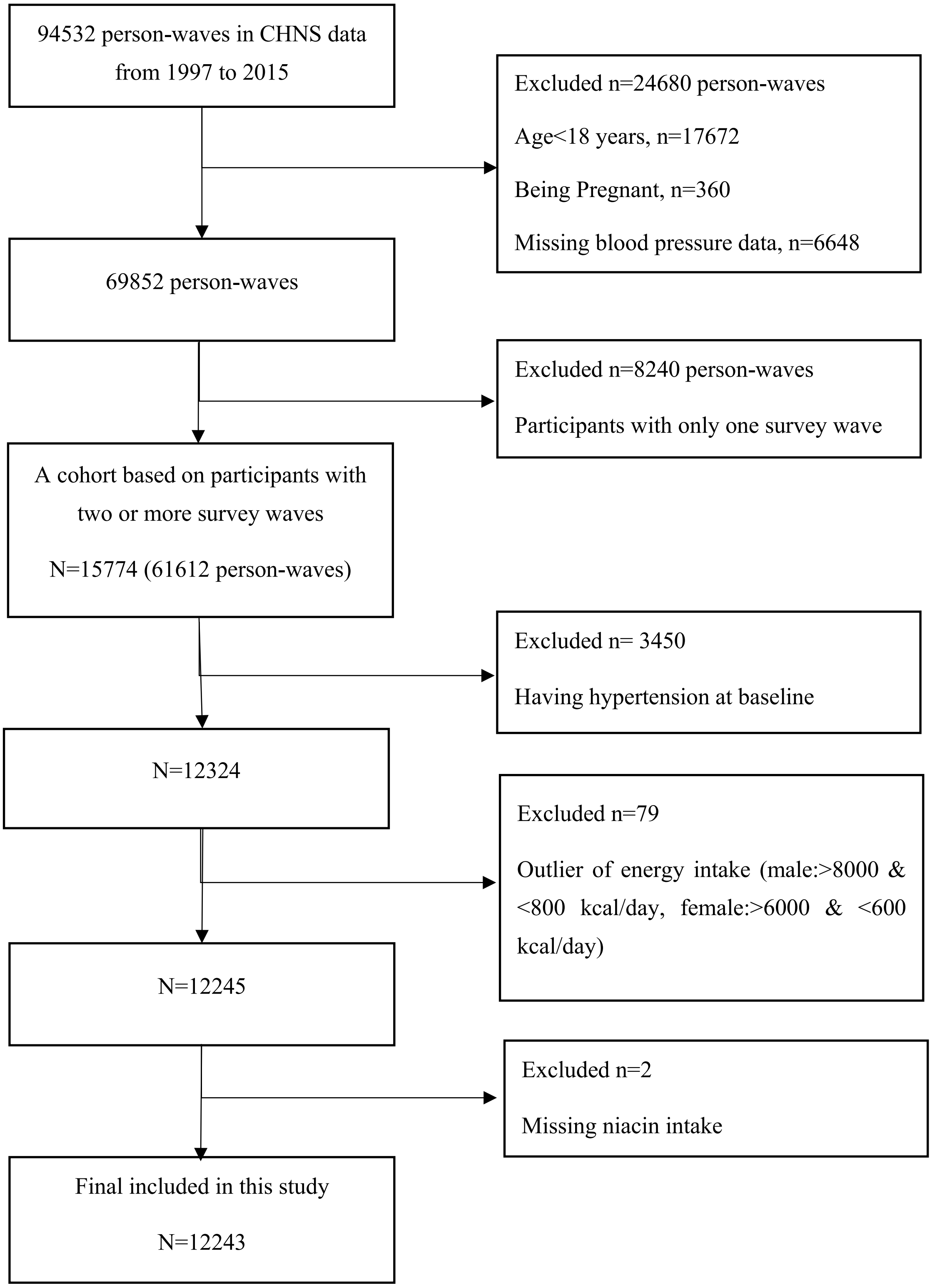

© 2021 Zhang Z et al. *JAMA Network Open.*

eFigure 2. Stratified analyses by potential effect modifiers for the association between dietary niacin intake and new-onset hypertension

Subgroup	Categories	Events (rate*)	Adjusted HR (95%CI)		Categories	Events (rate*)	Adjusted HR (95%CI)	P-interaction
BMI, kg/m^2								.47
<25	Q1-2	1754(43.8)	1.19(1.09,1.30)	≥25	Q1-2	582(75.8)	1.13(0.96,1.33)	
	Q3	736(32.3)	Ref		Q3	209(62.3)	Ref	
	Q4	712(39.8)	1.29(1.16,1.44)		Q4	283(88.0)	1.37(1.14,1.64)	
Smoking status								.07
No	Q1-2	1572(46.0)	1.23(1.11,1.36)	Yes	Q1-2	767(56.7)	1.10(0.97,1.25)	
	Q3	588(32.0)	Ref		Q3	358(45.7)	Ref	
	Q4	627(46.1)	1.43(1.27,1.60)		Q4	367(48.5)	1.14(0.99,1.33)	
Sodium to potassium intake ratio								.23
<2.8	Q1-2	967(50.4)	1.26(1.13,1.41)	≥2.8	Q1-2	1387(48.1)	1.12(1.00,1.25)	
	Q3	494(35.7)	Ref		Q3	458(36.6)	Ref	
	Q4	644(47.5)	1.33(1.18,1.50)		Q4	354(46.2)	1.30(1.13,1.50)	
Potassium intake, g/day								.17
<1.4	Q1-2	971(58.1)	1.24(1.08,1.42)	≥1.4	Q1-2	1383(44.2)	1.07(0.97,1.18)	
	Q3	296(38.9)	Ref		Q3	656(35.0)	Ref	
	Q4	202(54.3)	1.38(1.15,1.66)		Q4	796(45.5)	1.32(1.18,1.46)	
Energy intake, Kcal/day								.62
<2162.0	Q1-2	1062(49.7)	1.15(1.02,1.28)	≥2162.0	Q1-2	1292(48.5)	1.24(1.11,1.38)	
	Q3	483(36.9)	Ref		Q3	469(35.4)	Ref	
	Q4	431(47.3)	1.29(1.13,1.47)		Q4	567(46.9)	1.36(1.20,1.55)	
Fruits intake, g/day								.50
No	Q1-2	1675(67.9)	1.14(1.03,1.26)	Yes	Q1-2	679(29.1)	1.10(0.96,1.25)	
	Q3	606(50.9)	Ref		Q3	346(24.0)	Ref	
	Q4	626(66.5)	1.29(1.15,1.44)		Q4	372(31.5)	1.36(1.17,1.58)	
Vegetables intake, g/day								.02
<356.4	Q1-2	1202(45.5)	1.37(1.20,1.56)	≥356.4	Q1-2	1152(53.3)	1.14(1.03,1.26)	
	Q3	313(29.1)	Ref		Q3	639(41.1)	Ref	
	Q4	274(36.6)	1.29(1.10,1.53)		Q4	724(52.7)	1.30(1.16,1.45)	
Residence								.33
Rural	Q1-2	1722(48.0)	1.22(1.11,1.34)	Urban	Q1-2	632(52.1)	1.09(0.95,1.26)	
	Q3	617(33.8)	Ref		Q3	335(41.6)	Ref	
	Q4	597(44.9)	1.31(1.17,1.47)		Q4	401(50.6)	1.30(1.12,1.51)	
Education level								.89
Primary school or below	Q1-2	1295(60.6)	1.20(1.08,1.34)	Middle school or above	Q1-2	1002(39.5)	1.16(1.04,1.30)	
	Q3	460(43.6)	Ref		Q3	472(30.9)	Ref	
	Q4	384(59.9)	1.34(1.17,1.54)		Q4	600(41.4)	1.29(1.14,1.46)	

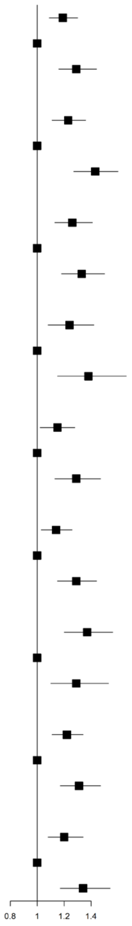

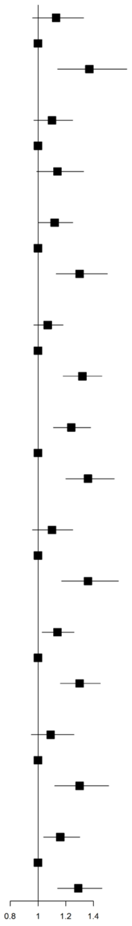

© 2021 Zhang Z et al. *JAMA Network Open.*

eTable 1. Characteristics of the included and excluded participants

Variables	Excluded*	Included
N, person-wave	14888	61612
Male, No. (%)	7528 (50.6)	29320 (47.6)
Age, years	42.0 ± 17.8	50.2 ± 15.0
Systolic blood pressure, mmHg	121.2 ± 18.1	123.7 ± 18.7
Diastolic blood pressure, mmHg	78.0 ± 10.9	79.4 ± 11.1
Body mass index, kg/m^2	23.2 ± 3.9	23.3 ± 3.4
Smoking status, No. (%)	4028 (29.2)	19191 (31.2)
Drinking status, No. (%)	4407 (32.2)	20242 (33.1)
Urban residence, No. (%)	6512 (43.7)	20058 (32.6)
Region, No. (%)		
East and central	8194 (55.0)	33599 (54.5)
Northeast and north	2798 (18.8)	12807 (20.8)
Southwest and south	3896 (26.2)	15206 (24.7)
Occupation, No. (%)		
Farmer	2455 (16.7)	17969 (29.5)
Worker	2044 (13.9)	5758 (9.4)
Unemployed	5659 (38.5)	23639 (38.8)
Other	4552 (30.9)	13600 (22.3)
Education, No. (%)		
Illiteracy	1435 (10.2)	12851 (21.6)
Primary school	1991 (14.1)	12448 (20.9)
Middle school	4991 (35.4)	18859 (31.7)
High school or above	5672 (40.3)	15329 (25.8)
Self-report diabetes, No. (%)	385 (2.6)	1638 (2.7)

Variables are presented as Mean ± SD or person-wave (%).

© 2021 Zhang Z et al. *JAMA Network Open.*

*Excluded from the analysis due to missing blood pressure data (6648 person-waves) and those with only one survey wave (8240 person-waves)

© 2021 Zhang Z et al. *JAMA Network Open.*

eTable 2. The association between dietary niacin intake and the risk of new-onset hypertension with further adjustment for waist/hip ratio, drinking status, sodium, fruits and vegetables intake

Niacin intake, mg/day	N	Events (rate*)	Crude model		Adjusted model†	
			HR (95% CI)	*P* value	HR (95% CI)	*P* value
Quartiles						
Q1 (<12.4)	3061	1188(51.7)	Ref		Ref	
Q2 (12.4-<14.3)	3060	1166(46.6)	0.90(0.83,0.97)	.009	0.92(0.84,1.01)	.07
Q3 (14.3-<16.7)	3061	952(36.2)	0.70(0.64,0.76)	<.001	0.81(0.74,0.89)	<.001
Q4 (≥16.7)	3061	998(47.0)	0.92(0.85,1.00)	.05	1.05(0.95,1.16)	.37
Categories						
Q1-2 (<14.3)	6121	2354(49.0)	1.36(1.26,1.47)	<.001	1.17(1.08,1.27)	<.001
Q3 (14.3-<16.7)	3061	952(36.2)	Ref		Ref	
Q4 (≥16.7)	3061	998(47.0)	1.32(1.21,1.44)	<.001	1.29(1.17,1.42)	<.001

*Incident rate is presented per 1000 person-years of follow-up.

†Adjusted for age, sex, body mass index, waist/hip ratio, smoking status, drinking status, systolic blood pressure, diastolic blood pressure, region, education, occupation, as well as energy, sodium, fruits and vegetables intake and sodium to potassium intake ratio.

© 2021 Zhang Z et al. *JAMA Network Open.*

eTable 3. The association between dietary niacin intake and the risk of new-onset hypertension with exclusion of participants from the three autonomous cities

Niacin intake, mg/day	N	Events (rate[*])	Crude model		Adjusted model[†]	
			HR (95% CI)	*P* value	HR (95% CI)	*P* value
Quartiles						
Q1 (<12.3)	2725	1100(51.6)	Ref		Ref	
Q2 (12.3-<14.2)	2724	1067(45.9)	0.88(0.81,0.96)	.004	0.94(0.86,1.02)	.150
Q3 (14.2-<16.4)	2725	910(36.3)	0.70(0.64,0.76)	<.001	0.84(0.77,0.93)	<.001
Q4 (≥16.4)	2725	945(44.8)	0.87(0.80,0.95)	.002	1.07(0.97,1.18)	.16
Categories						
Q1-2 (<14.2)	5449	2167(48.6)	1.34(1.24,1.45)	<.001	1.14(1.05,1.24)	.001
Q3 (14.2-<16.4)	2725	910(36.3)	Ref		Ref	
Q4 (≥16.4)	2725	945(44.8)	1.25(1.14,1.37)	<.001	1.27(1.16,1.39)	<.001

[*]Incident rate is presented per 1000 person-years of follow-up.

[†]Adjusted for age, sex, body mass index, smoking status, systolic blood pressure, diastolic blood pressure, region, education, occupation, as well as energy intake and sodium to potassium intake ratio.

© 2021 Zhang Z et al. *JAMA Network Open.*

eTable 4. The association between dietary niacin intake and different components of new-onset hypertension

Niacin intake, mg/day	N	Events (rate*)	Crude model		Adjusted model†	
			HR (95% CI)	*P* value	HR (95% CI)	*P* value
**Physician-diagnosed hypertension**						
Categories						
Q1-2 (<14.3)	6084	427(8.9)	1.19(1.01,1.42)	.04	1.02(0.85,1.21)	.86
Q3 (14.3-<16.7)	3041	195(7.4)	Ref		Ref	
Q4 (≥16.7)	3037	212(10.0)	1.34(1.11,1.63)	.003	1.33(1.09,1.63)	.005
**Use of antihypertensive treatment**						
Categories						
Q1-2 (<14.3)	6088	271(5.6)	1.24(1.00,1.54)	.05	1.08(0.86,1.36)	.49
Q3 (14.3-<16.7)	3042	119(4.5)	Ref		Ref	
Q4 (≥16.7)	3038	143(6.7)	1.47(1.15,1.88)	.002	1.46(1.13,1.88)	.004
**SBP ≥140 and/or DBP ≥90 mmHg**						
Categories						
Q1-2 (<14.3)	6121	2175(45.3)	1.38(1.27,1.49)	<.001	1.20(1.11,1.30)	<.001
Q3 (14.3-<16.7)	3061	868(33.0)	Ref		Ref	
Q4 (≥16.7)	3061	912(43.0)	1.33(1.21,1.46)	<.001	1.32(1.20,1.45)	<.001

*Incident rate is presented per 1000 person-years of follow-up.

†Adjusted for age, sex, body mass index, smoking status, systolic blood pressure, diastolic blood pressure, region, education, occupation, as well as energy intake and sodium to potassium intake ratio.

© 2021 Zhang Z et al. *JAMA Network Open.*
